# A Clerical Publican

**Published:** 1891-04-25

**Authors:** 


### The Doctor's Armchair.

### A CLERICAL PUBLICAN.

Hampton Lucy is not altogether unknown to readers of Shakespeare—a very extensive class—but it promises to be known soon to a number of persons who can hardly be credited with much Shakespearian lore, viz., publicans and total abstainers. The present rector of Hampton Lucy is the Reverend Osbert Mordaunt; he is the proprietor of the one public-house of the village; and he "runs the business" himself. He does not, of course, live at the inn, and draw the beer; nor does he wisp down the horses, and touch his hat to the grudging farmers when they give him sixpence for what they shrewdly guess has been a very small feed of corn. Mr. Mordaunt lives at the rectory, and delegates his publican's duties to a suitable satrap. It is a refreshing little picture which the rector presents in his brief pamphlet; almost as refreshing as a glass of his excellent "Hampton Arms" beer would be to a Shakespeare pilgrim on a dusty midsummer day.

Mr. Mordaunt is that curious *bête noire* to the fanatical total abstainer, a moderate man. He does not believe in tying up his people's hands lest they should pick and steal their neighbours' potatoes. He thinks it better that they should have the chance of buying genuinely good potatoes at a moderate price, and of eating as many as they need, and no more. Odd as the statement may sound, this village rector "runs" the public-house in the interests of temperance; and, odder still, as some will think, he has found his venture entirely successful. This common sense and non-fanatical view of the situation is altogether worthy of the soil out of which our broad and generous Shakespeare grew. The longest way round is often the shortest way home, though one can seldom persuade a fool to try the detour.

This is a scientific age, and the rector of Hampton Lucy has made an experiment in Sociology. He does not call it by any such fine name himself, because he knows better; but we call it so, because we know that some of our readers are so exclusively and transcendantly scientific that if we do not present to them Mr. Mordaunt's wholesome little dish under a learned name and in a little frill of red tissue paper, they will turn up their scientific noses at it and refuse to touch it, even with an alpenstock. A "Sociological experiment," therefore, is what our high scientific readers are invited to consider.

The rector has been the inspiring genius of his own village inn for about sixteen years. He has, therefore, had ample time in which to prove the merits of his system of business. For he has a system, though by no means an elaborate one. His leading principles are two: the first is that he sells only pure beer, and the second that he has no interest whatever in the profits. But why then does he carry on the business? asks our "Sociologist" of the "survival-of-the-fittest" school. He carries on the business because this particular public-house is the only one in the village; and if it were closed, it is pretty certain that another would soon be opened: another over which neither he nor any other sobriety-loving person might have the very least control. The question which the rector really had to decide was whether he should treat his villagers like grown-up men who had the sense to take care of themselves, or like wilful children who could not be trusted beyond the range of the schoolmaster's eye. He decided, as the new Archbishop of York would have decided, that at any rate he would make his people free, and, as far as lay in his power, freely sober.

"Pure beer" was the rector's first principle of business. This, it will be seen, differs from that of the famous Yorkshire political economist: his first principle was "to make something out of nothing," or, as he more indigenously expressed it, "To mak summat aht o' nowt." "Pure beer," asks the sceptical abstainer; "what may that be, and where may it be found?" Our Shakespeare-minded rector suggests two common-sense answers to that question: He might have brewed the beer for himself, in which case he would have known exactly what its standard of purity was; or it was open to him to do exactly what he did do—that is, to try all the brewers in the neighbourhood until he found one who could be trusted to supply a good honest article. He seems, as a matter of fact, to have had no difficulty, and that for two reasons: first, because Englishmen are naturally inclined to be honest; and, secondly, because they are not such fools as not to know that a good article commands a good price and a steady market. Is there, then, no impure beer in this country? Plenty! But we make no doubt whatever that there is much more which is both pure and wholesome.

What about the manners of the villagers under this rector-publican system? Two good results have followed. "I have reason to believe," says the rector, "that on account of the liquor being pure and wholesome, and therefore satisfying, much less is drunk than formerly." This is all clear gain to the wives and families of the consumers, and it is equally clear gain to the health and life of the men themselves. In the old days, before the public-house changed hands, drunkenness—though not worse in Hampton Lucy than elsewhere—was quite common. "I am thankful to say now," says the rector, that cases "are comparatively rare, and seldom occur except people have come in from other places the worse for liquor, and have been accidentally served with more. Of course, if such a condition is perceived they are declined any at all. The usual public-house hours are observed, and no limit as to the quantity supplied to sober people is ever attempted; but no credit is allowed."

"What about the balance-sheet?" asks the man of business. Does this amiable and original rector make it pay, or does his satrap, like other satraps, get rich himself at the expense of the imperial exchequer? The satrap seems to make a very comfortable living out of those parts of the public-house business which are not connected with drink selling. But the beer itself makes a considerable profit, thirty pounds in fact, and this is devoted to the village charities. The rent of the inn, which is twenty-five pounds, pays the salary of the church organist; and an uncommonly lucky organist he is to have such a salary in a village. The rector, it is evident, gets nothing whatever out of the business. He gives his intelligence and his conscience to its supervision; and his all sufficient reward is the sobriety, comfort, and health of his parishioners.

It was said at the beginning that a "sociological experiment," extending over sixteen years, had been made at Hampton Lucy; and that it had been made in the interests of temperance. The rector appears to address his pamphlet principally to temperance reformers. Do not be so anxious, he says in effect, to stamp out everything and everybody connected with the liquor trade. You set yourselves a quite impossible task when you essay its complete destruction. Human nature is against you: the human nature of the seller on the one hand, and of the buyer on the other. The Germans are a sober people; and why? Because they drink pure beer. Set yourselves the duty of regulating the British brewing trade, and of ensuring for the British customer a pure and wholesome beer. This is a task which you may accomplish; and that without very much difficulty. Why beat out your brains and tear out your hearts in attempting that which can never succeed? The small religion of the fanatic raises a sect, and sometimes an exceedingly disagreeable one; the large religion of the man of sense founds a church—a church whose wholesome example and doctrine is the antiseptic leaven which purifies the body politic. Temperance is a department of religion which all men admire and believe in; even total abstinence is good and necessary for dyspeptic minds whose spiritual digestion is injured by strong drink. But the wise physician is not he who has one and the same rule for all; but he who has wholesome principles from which each free and intelligent man may draw his own suitable and necessary rules.

